# Expression of concern: ‘Long non-coding RNA LINC00346 contributes to cisplatin resistance in nasopharyngeal carcinoma by repressing miR-342-5p’ (2020), by Cui Z *et al.*

**DOI:** 10.1098/rsob.230304

**Published:** 2023-08-31

**Authors:** Open Biology editorial team

Following the publication of ‘Long non-coding RNA LINC00346 contributes to cisplatin resistance in nasopharyngeal carcinoma by repressing miR-342-5p’, the Editorial team have concerns regarding the validity of the data used in this study.

We are issuing an expression of concern here and investigating this as a matter of urgency; we will notify readers as to the results of our investigation as soon as possible.

